# Evaluation of aflatoxin contamination in protein-rich pulses using a GFP-expressing *Aspergillus flavus* strain

**DOI:** 10.3389/fmicb.2025.1587035

**Published:** 2025-05-29

**Authors:** Emily H. Branstad-Spates, Christine M. Sickler, Matthew D. Lebar, Carol Carter-Wientjes, Kanniah Rajasekaran

**Affiliations:** Food and Feed Safety Research Unit, USDA-ARS Southern Regional Research Center, New Orleans, LA, United States

**Keywords:** aflatoxin contamination, cyclopiazonic acid, kernel screening assay, green fluorescent protein, pulses

## Abstract

**Background:**

Mycotoxigenic fungi pose significant threats to food safety and marketability. Crop-specific differences in susceptibility to these fungi can influence contamination levels.

**Objectives:**

The resistance or susceptibility of protein-rich pulse crops—chickpeas (*Cicer arietinum* L. cv. CDC Frontier), lentils (*Lens culinaris* Medik cv. Eston), peas (*Pisum sativum* L. cv. LeRoy), and corn (*Zea mays* L. cv. H97C) to infection by *Aspergillus flavus* were evaluated using a kernel screening assay (KSA).

**Methodology:**

*A. flavus* strain 70 (AF-70) expressing green-fluorescent protein (GFP) was used to quantify fungal spread and mycotoxin production. Fungal infection and toxin levels, including aflatoxins (AFB_1_, AFB_2_), cyclopiazonic acid (CPA), and α-aflatrem, were monitored at 2-day intervals over a 10-day period post inoculation.

**Results:**

Although all seeds were infected by *A. flavus*, corn produced significantly higher levels of AFB_1_ and AFB_2_ compared to pulses. However, pulses accumulated relatively higher levels of CPA and α‑aflatrem.

**Conclusion:**

While pulses may be less susceptible to aflatoxin contamination than corn, the elevated concentrations of CPA and α‑aflatrem underscore the need for further toxicological evaluation and mechanistic studies. Future research should explore the underlying resistance mechanisms from field to storage to better ensure crop safety.

## 1 Introduction

Increasing human population and depletion of natural resources in recent decades have become critical issues that need to be addressed globally (Quintieri et al., 2023). The global population is projected to reach 9.7 billion by 2050, up from the current 8.0 billion, thus adding approximately 1.7 billion people over the next 26 years (United Nations Department of Economic and Social Affairs, 2022). Consequently, the demand for healthy, nutritious, and sustainable food and feed products is paramount. Current food and feed systems, particularly livestock-based meat production, would benefit from incorporating plant-rich protein sources like pulses (i.e., beans, chickpeas, lentils, etc.). This would create a synergistic balance to feed the growing population and reduce environmental impacts through decreased water and land usage (Heinke et al., 2020; Godfray et al., 2018). The plant-based protein industry is rapidly expanding and is projected to become a $27 billion industry by 2030 (Singh et al., 2024). Including pulses would aid in diversifying cropping systems, providing a sustainable nutrition source, and developing a resilient food and feed supply chain for the growing population (Singh et al., 2024).

Chickpeas (*Cicer arietinum* L.), lentils (*Lens culinaris* Medik), and peas (*Pisum sativum* L.) are cultivated pulse crops grown worldwide, belonging to the *Fabaceae* (*Leguminosae*) family (Henchion et al., 2017; Quintieri et al., 2023). Currently, these pulses offer a good balance between sustainability and nutritional value, providing high dietary protein, fiber, and energy content (Acuña-Gutiérrez et al., 2022). The Dietary Guidelines Advisory Committee recommends a plant-based diet to reduce cholesterol, improve muscle mass, maintain bone health, address obesity, and meet protein requirements more effectively than animal-based proteins (Gavrilova et al., 2020; George et al., 2020; Singh et al., 2024). Protein concentrations for chickpeas, lentils, and peas range from 12.6–30.6%, 20.6–31.4%, and 21.2–32.9%, respectively (Dahl et al., 2012; Wood and Grusak, 2007; Yadav et al., 2007). In comparison, the average protein content of animal-based proteins, excluding fish and insects, is 22.0% (Day et al., 2022). Introducing pulses into diets may prevent various diseases, enhance global food and feed security, and provide high protein content alongside traditional animal-meat products or in formulations for meat alternatives (Hertzler et al., 2020; Massawe et al., 2016; Quintieri et al., 2023). Despite being labeled as “underutilized” legume crops, pulses are widely used in many countries. These plant-rich protein sources need to be evaluated for food and feed safety and quality due to their inclusion in human and livestock diets. Pulses are excellent candidates for developing novel food and feed products, including meat-based alternatives (Acuña-Gutiérrez et al., 2022; Gräfenhahn and Beyrer, 2024; Rebello et al., 2014).

Contamination of grains and pulses by fungi, and subsequently mycotoxins, can occur at any stage of the supply chain: in the field, at harvest, during transportation, and storage (Begum and Samajpati, 2000). Aflatoxins, secondary metabolites produced by *Aspergillus* spp., contaminate a variety of food and feed crops globally, including well-studied crops such as corn, cottonseeds, peanuts, and tree nuts pre- and post-harvest (CAST, 2003). Aflatoxin B_1_ (AFB_1_), a potent mutagenic and carcinogenic mycotoxin produced by *Aspergillus flavus* and *A. parasiticus*, has been extensively studied in various food and feed sources (Kumar et al., 2021). However, there is limited literature on the susceptibility of pulses to *Aspergillus* spp. and aflatoxin production (including strains B_1_ and B_2_) (Acuña-Gutiérrez et al., 2022). Currently, there are no specific regulatory limits for aflatoxin in pulses; however, in the US, corn has a 20-ppb (parts per billion) limit for general commerce (FDA, 2025). Chickpeas have been documented as susceptible to fungal pathogens, including *Aspergillus* and *Fusarium* species, primarily due to poor field conditions, mechanical damage during harvest, inadequate transportation, processing issues, and poor storage conditions (Donato et al., 2022; Ramirez et al., 2018). While aflatoxin has been reported in lentils in Iran and Egypt, the prevalence was low, with mean levels below the limit of detection (LOD) (Ahmadi et al., 2022; El-Maraghy, 1988). Conversely, in Bangladesh, aflatoxin levels in lentils exceeded the U.S. maximum regulatory limit of 20 ppb for human food consumption (Roy et al., 2013). Peas (*Pisum sativum* L.) have shown high resistance to aflatoxin formation, with studies indicating no detectable toxin from pulses infected with *A. flavus* (El-Kady et al., 1996).

With limited data on the accumulation of aflatoxin in pulses such as chickpeas, lentils, and peas, more studies are needed to understand the effects of infection, given the increasing demand for protein-rich plant sources for dietary consumption and the need to address public health concerns and sustainability of food supply systems. This study utilized *Aspergillus flavus* AF-70, a toxin-producing strain expressing a green fluorescent protein (GFP), to assess fungal growth and the accumulation rates of AFB_1_, AFB_2_, cyclopiazonic acid (CPA), and α-aflatrem in chickpeas, lentils, peas, and corn over a 10-day period with 2-day interval sampling post-inoculation. The objective was to understand fungal infection patterns, colonization, growth, and aflatoxin accumulation in protein-rich pulses using a kernel screening assay (KSA). The hypothesis was that under identical infection pressure by *Aspergillus flavus*, protein-rich pulse crops, specifically chickpeas, lentils, and peas, accumulate lower levels of aflatoxins compared to corn.

## 2 Materials and methods

### 2.1 Fungal strains and growth conditions

Aflatoxigenic *A. flavus* 70-GFP (AF-70 GFP) was grown in the dark at 31°C on a 2X V8 medium (10% V8 juice, 2% agar, pH 5.2) (Rajasekaran et al., 2008). Spores from 7-d old cultures were suspended in 0.02% Triton X-100, and the conidial concentration was determined with a hemocytometer and adjusted to 3.0 × 10^6^ conidia/mL. In AF-70 GFP, GFP is produced under control of the constitutively expressed *A. nidulans* glyceraldehyde phosphate dehydrogenase (*gpdA*) gene promoter inserted into *niaD* (Rajasekaran et al., 2008).

### 2.2 Plant-rich protein sources

Chickpeas [*Cicer arietinum* L. cv. CDC Frontier], lentils [*Lens culinaris* Medik cv. Eston], and peas [*Pisum sativum* L. cv. LeRoy] were provided by USDA-ARS laboratories (Dr. Clarice J. Coyne and Dr. Marilyn Warburton, Western Regional Plant Introduction Station, Washington State University, Pullman, WA). The seed source location and year for chickpeas, lentils, and peas were Central Ferry, WA, and Richland, MT, in years 2022, 2019, and 2023, respectively. These varieties were selected due to germplasm resources provided by USDA-ARS. A non-GMO, yellow, dent-corn hybrid [*Zea mays*], H97C (Hybrid85, Omaha, NE, United States) was purchased from corn produced for growers seed in 2022. Corn was included as a positive control in this study. All botanical terms in this manuscript are according to Kiesselbach (1999) and Allaire and Brady (2008).

### 2.3 Kernel screening assay

**Figure 1:**
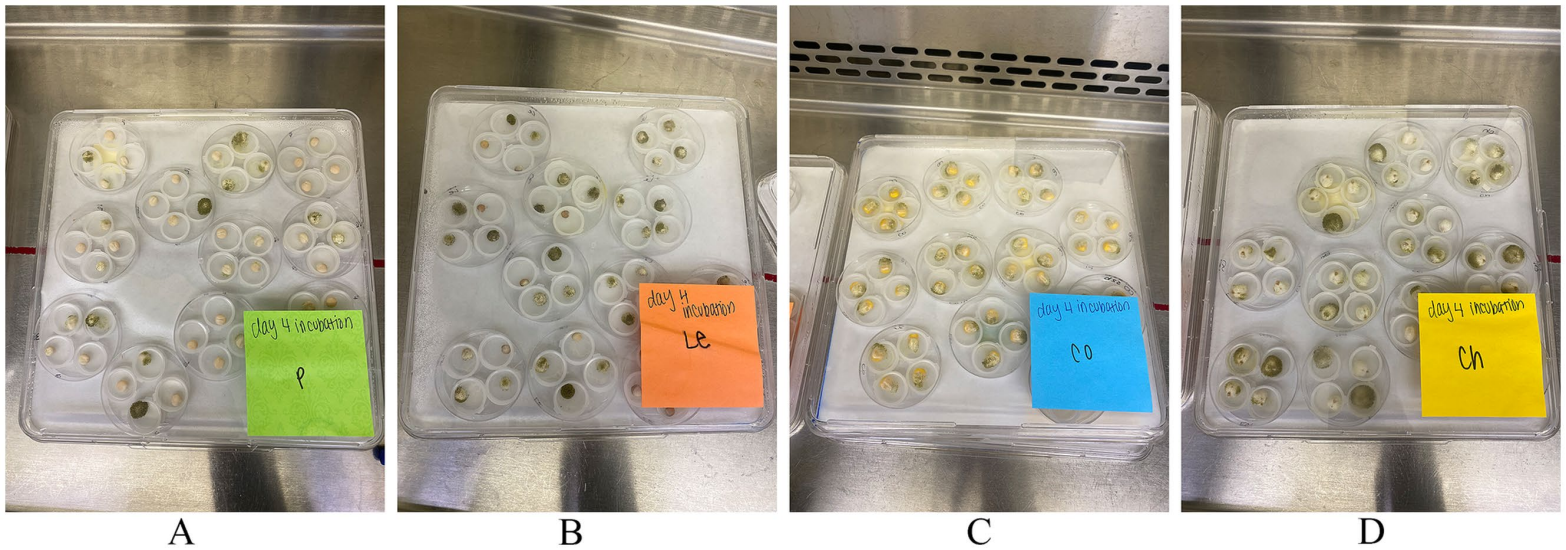
A typical kernel screening assay (KSA) set up. Each culture dish contains 4 seeds, constituting a replication, with 9 total replicates for analysis and 2 replicates for imaging. Seeds showcase *Aspergillus flavus* infection on day 4 of **(A)** peas, **(B)** lentils, **(C)** corn, and **(D)** chickpeas.

Undamaged lentil, corn, pea, and chickpea seeds were surface sterilized with 70% ethanol and subjected to a KSA (Brown et al., 1993; Brown et al., 1995; Rajasekaran et al., 2013). The same procedures developed for corn were used for pulses to determine *A. flavus* infection, as there was a lack of published methods specifically for pulses. Surface sterilized seeds were briefly immersed in a 3.0 × 10^6^ conidial inoculum and placed in plastic vial caps (20 mm diameter, 6 mm height). The same procedure was applied for day 0 seeds but were not treated with *A. flavus* suspension. Four caps were placed in an open-tissue culture dish (60 × 15 mm; Becton Dickinson, CO., Oxnard, CA, United States), representing one experimental unit. Culture dishes were placed side by side in a clear tray (243 × 243 × 18 mm, Nunc bioassay dish; Thomas Scientific, Swedesboro, NJ, USA) lined with 3 mm chromatography paper (Whatman International, Maidstone, UK) (Figure 1). The lid was placed on top of the tray but was not sealed. High humidity (>95% RH) was maintained by adding 25 mL of sterile deionized water to the trays. Seeds were incubated in the dark at 31°C and sampled at day 0, 2, 4, 6, 8, and 10 after inoculation with *A. flavus*.

### 2.4 GFP quantitation

Chickpeas, lentils, peas, and corn were ground and homogenized using a SPEX SamplePrep Geno/Grinder 2010 (1740 rpm, 1.5 min; Cole-Parmer, Vernon Hills, IL, United States), with 3/8″ stainless steel balls. The samples were stored at −80°C until analysis, where seeds were diluted in 1.0 mL Sorenson’s phosphate buffer (pH 7.0) and centrifuged at 10,000 rpm for 15 min. Supernatants were then analyzed for GFP using a BioTek Synergy Neo2 (Agilent, Santa Clara, CA) with excitation at 485 nm and emission at 535 nm. Relative fluorescence units were used for statistical analyses and normalized as % values relative to the highest data point.

### 2.5 Aflatoxin, cyclopiazonic acid, and α-alfatrem extraction and analysis

Following the growth of fungal strains on seeds, AFB_1_, AFB_2_, CPA, and α-aflatrem were extracted for analysis. The materials were ground in 15 mL polycarbonate vials (OPS Diagnostics, Lebanon, NJ, United States) with a SPEX SamplePrep Geno/Grinder 2010 (1,740 rpm, 1.5 min; Cole-Parmer, Vernon Hills, IL, United States), and 25 mg of sample material was weighed with 500 mL of 100% methanol (MeOH). Samples were shaken overnight on a shaker table at room temperature (22°C) and 210 RPM. The extracts were filtered through cotton plugs, and the filtrates were concentrated using a Savant speedvac (Thermo Scientific). Each extract was redissolved in methanol (1 mL), particulates were removed via centrifuge, and the supernatant was analyzed using a Waters (Milford, MA, United States) Acquity Ultra Performance Liquid Chromatography (UPLC) system (40% methanol in water, BEH C18 1.7 μm, 2.1 mm × 50 mm column) using fluorescence detection (excitation: λ = 365 nm, emission: λ = 440 nm). Samples were diluted to 10-fold if the aflatoxin signal saturated the detector. An analytical standard (Sigma-Aldrich) was used to identify and quantify AFB_1_ and AFB_2_. Aflatoxin content was expressed in ng AFB_1_/g and AFB_2_/g seed sample (ppb).

To assess the presence of CPA and α-aflatrem, the re-dissolved, centrifuged extracts were diluted 10-fold and analyzed on a Waters Acquity UPLC and Xevo G2 XS QTOF mass spectrometer (MS) as previously reported (Moore et al., 2022), briefly: the MS was equipped with a Z-spray ionization source running in ESI + mode using Waters MassLynx 4.2 software Separation was achieved with a gradient solvent system (A: 0.1% formic acid in water; B: 0.1% formic acid in acetonitrile) on a Waters BEH C18 1.7 μm, 2.1 × 50 mm column: 5% B (0–1.25 min.), to 25% B (1.25–1.5 min.), to 100% B (1.5–5.0 min.), then 100% B (5.0–7.5 min.), followed by column equilibration at 5% B (7.6–10.1 min.). Data were analyzed on Waters UNIFI 1.9.4 software using the “Quantify Assay Tof 2D” analysis method with lock mass corrected by UNIFI. CPA and α-aflatrem was purchased from Sigma-Aldrich (Sigma-Aldrich, St. Louis, MO, United States) and used for quantification. CPA and α-aflatrem content were expressed in ng CPA/g per sample (ppb) and ng α-aflatrem/g per sample (ppb).

### 2.6 Microscopy

At each sampling time frame, eight seeds were randomly chosen and photographed using a Nikon-SMZ25 research stereomicroscope (Nikon Instruments, Melville, NY, United States) equipped with an Andor Zyla 4.2 sCMOS Digital Camera (Nikon Instruments, Melville, NY, United States) including fluorescence and bright field images of AF-70 GFP. The seeds were then divided evenly for use in aflatoxin analysis and GFP quantitation. A minimum of 9 replicates (4 seeds each) were used for each day of sampling. The seed exteriors were cleaned with 9 mL of deionized water and vortexed for 15 s to remove spores on the seed pericarps for counting and stored at 4.0°C. All seeds were photographed both externally and internally with a horizontal cross-section following methods published by Rajasekaran et al. (2013).

### 2.7 Spore counts on seed coats

The seeds were placed in 15 mL centrifuge tubes with a plug seal cap (Corning Inc., Corning, NY, United States) with 9 mL of deionized water, vortexed for 15 s, and removed to collect external mycelia for spore counts. The samples were stored at 4.44°C until analysis, where samples were vortexed for 5 s, 10 μL of liquid was pipetted into disposable hemocytometer slides containing 4 replicates per sample and analyzed using an Olympus Cell Counter model R1 (Olympus Life Sciences, Waltham, MA, United States). The seeds were externally cleaned with Kimtech Wipes (Kimtech Science, Vaughan, Ontario), deionized water, and placed in 15 mL polycarbonate vials (OPS Diagnostics, Lebanon, NJ, United States) stored at −80°C until further analysis.

### 2.8 Statistical analysis

Average AF-70 GFP fluorescence, AFB_1_ and AFB_2_ values, external spore counts, CPA, and α-aflatrem from a minimum of nine replicates per days 0, 2, 4, 6, 8, and 10 were subjected to two-way ANOVA with Geisser–Greenhouse correction, with Dunnett’s multiple comparison to test for simple effects within rows in GraphPad Prism (version 10.2.0) software (GraphPad Software Inc., La Jolla, CA, USA). The fixed effects were time and plant type, and random-effects were aflatoxin levels. Corn kernels were used as control for comparison of infection and contamination versus pulses. To investigate the relationship between aflatoxin and AF-70 GFP expression, a correlation analysis was performed using Pearson correlation coefficients. Statistical significance for treatment effects were declared at *p* ≤ 0.05. Trends are discussed at *p* ≤ 0.10. All data is presented as means ± the standard error of the mean (SEM) unless stated otherwise.

## 3 Results

### 3.1 Fungal entry, infection, and colonization in seeds

**Figure 2:**
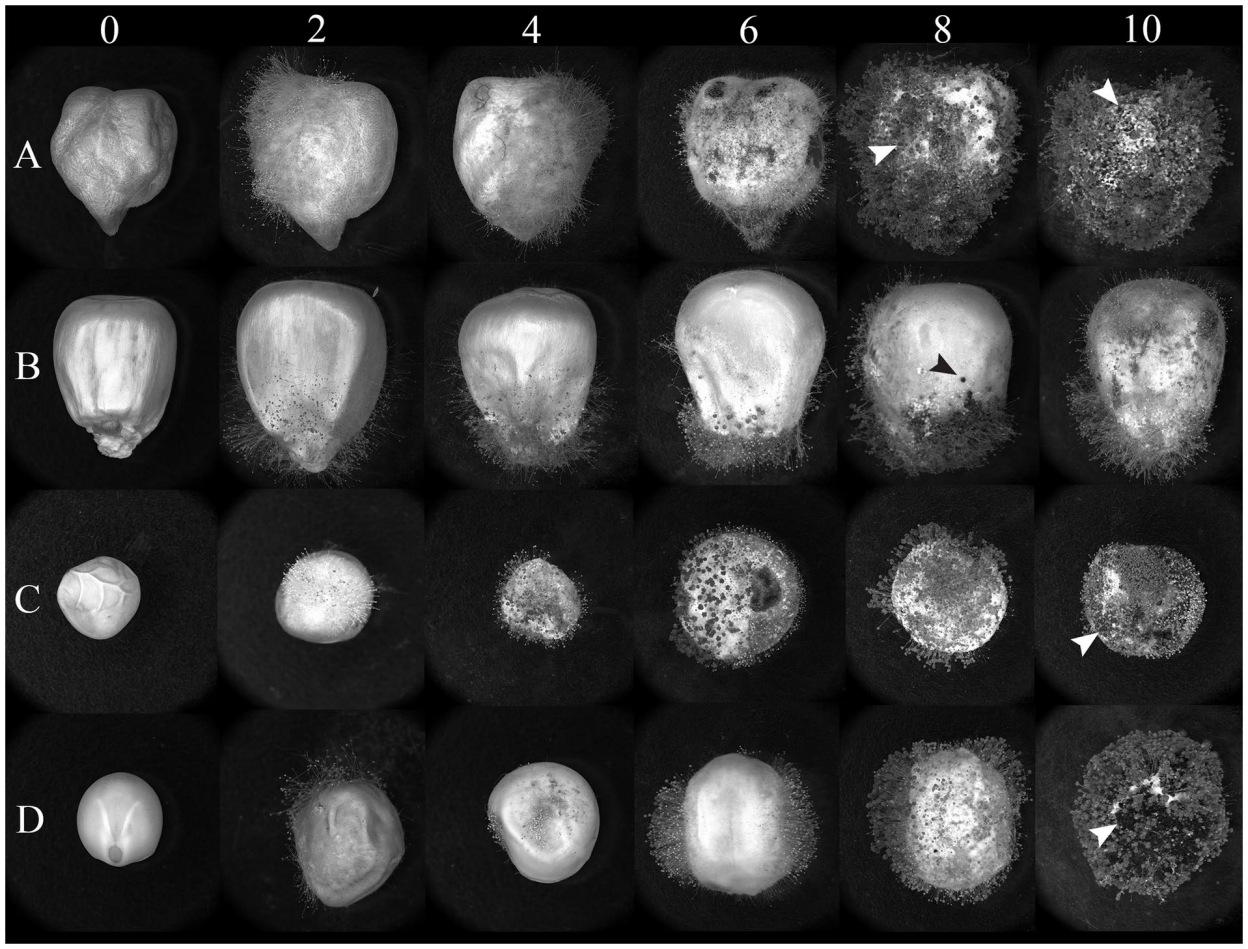
Light micrographs of **(A)** chickpeas, **(B)** corn, **(C)** peas, and **(D)** lentils over time (days) after inoculation with AF 70-GFP. Visual photographs are taken on a 2-day interval from day 0 to day 10. The arrows represent sclerotia formation across seeds.

Undamaged seeds from four protein-rich plant sources (chickpeas, lentils, peas, and corn) were inoculated with AF-70 GFP conidial suspension, and the fungus was allowed to colonize the seeds under high humidity conditions (Figure 1). Infected seeds were observed under a stereo light microscope and photographed at regular intervals for each day (0, 2, 4, 6, 8, and 10), as shown in Figure 2. Mycelia were observed on the seed coats 2 days after inoculation in all seeds. The first visible infection for corn kernels was at the pedicel, whereas pulses had visible infection around the entire seed coat on day 2 (Figure 2). By day 4, lentils exhibited visible swelling and on day 6, peas and chickpeas swelled due to internal fungal growth (Figure 2). Sclerotia development was noted only on lentils by day 4 and was present on all seeds and kernels by day 6. On day 10, the entire endosperm was colonized to the fungus across all seeds. Future studies will separate out the endosperm and embryo to determine where fungal growth is localized.

**Figure 3:**
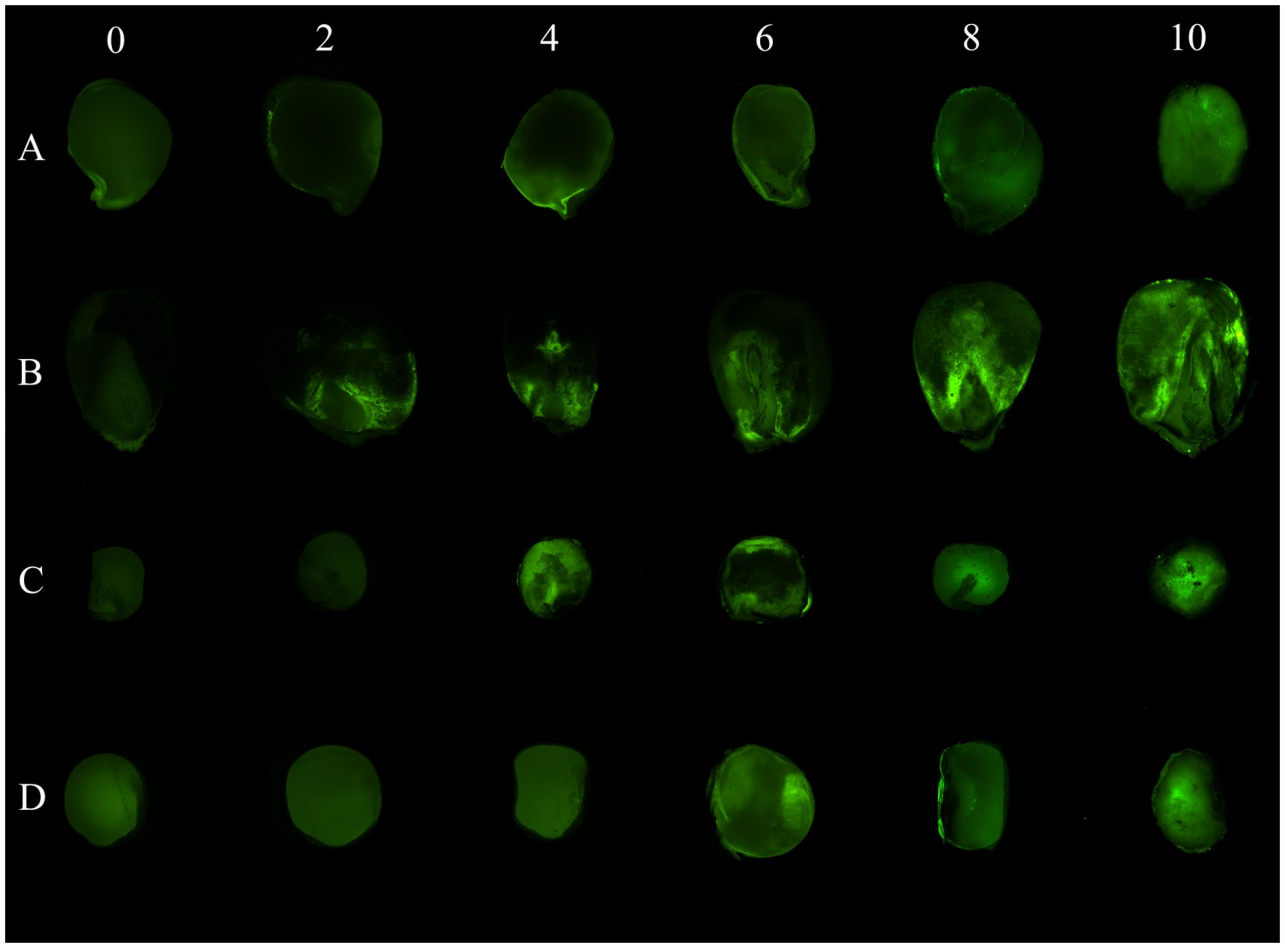
AF 70-GFP fluorescence in longitudinal sections of **(A)** chickpeas, **(B)** corn, **(C)** peas, and **(D)** lentils over time (in days) after inoculation. Visual photographs are taken on a 2-day interval from day 0 to day 10.

Fluorescence due to AF-70 GFP growth inside the seeds of chickpeas, peas, lentils, and corn was detected in longitudinal sections (Figure 3). A typical progression of AF-70 GFP fluorescence was observed in corn kernels, where entry was first observed by near the corn pedicel (base), which spread throughout the endosperm and embryo by day 10. Fluorescence in chickpea micropyles was also observed. Production of sclerotia was observed inside all seeds, including corn kernels, by day 8; however, the sclerotia did not fluoresce. The path of fungal spread was more evident in corn than the other pulses (Figure 3).

### 3.2 External *Aspergillus flavus* spore counts

**Figure 4:**
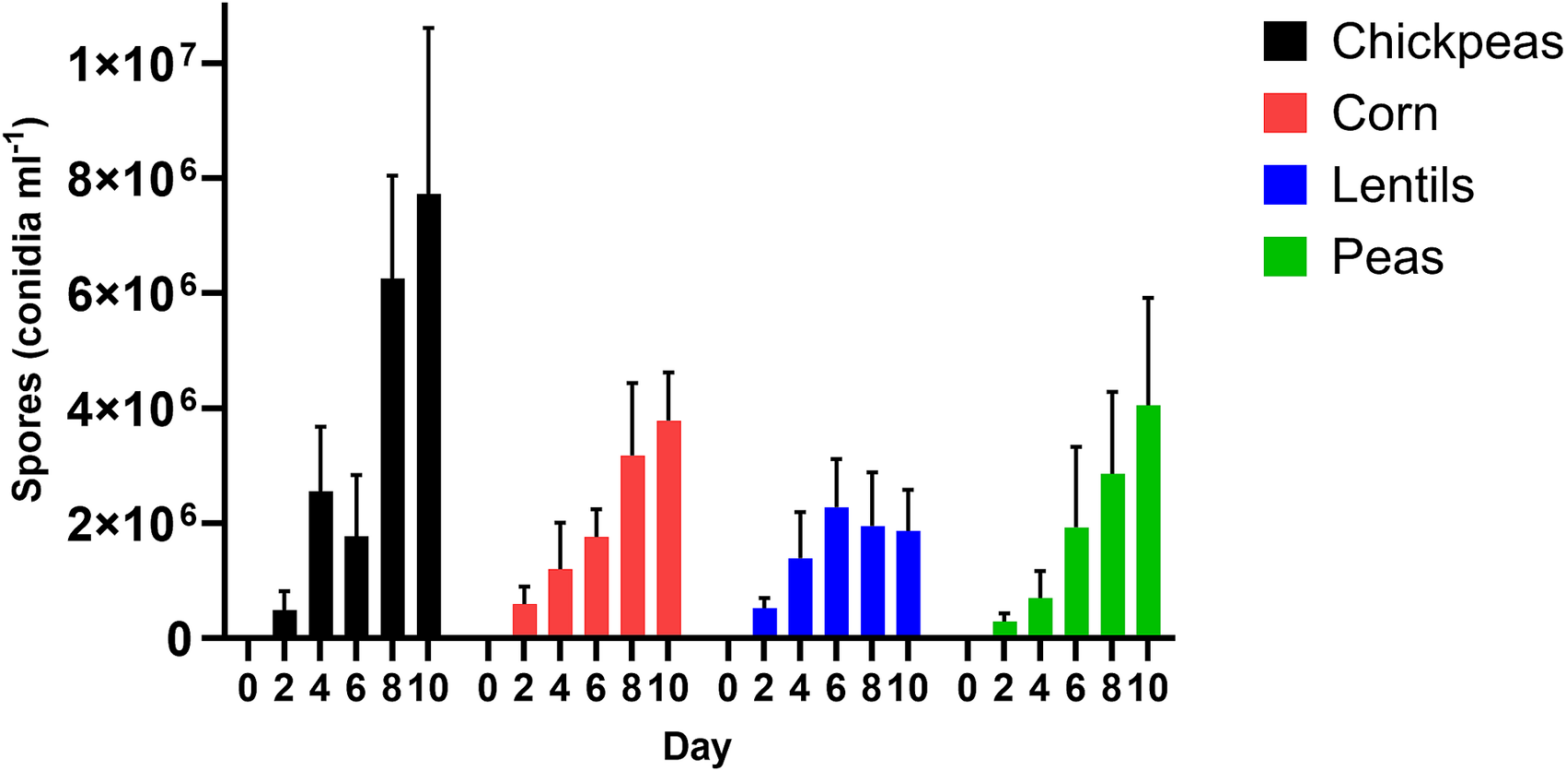
*Aspergillus flavus* spores (conidia ml^−1^) on seed coats of chickpeas, corn, lentils, and peas over a 10-day interval period.

Spores were collected from seed coats of chickpeas, lentils, peas, and corn for the respective day-intervals of 2, 4, 6, 8, and 10 (Figure 4); day 0 was not measured due to surface seed sterilization. Chickpeas resulted in the greatest conidial production by *A. flavus* spores (3.76 × 10^6^ ± 1.38 × 10^5^ conidia mL^−1^), whereas lentils supported the least conidial production (1.60 × 10^6^ ± 3.04 × 10^5^ conidia mL^−1^) (*p* = 0.24; Supplementary Table 1). *Aspergillus flavus* spore production (conidia mL^−1^) increased each day on chickpeas, corn, and peas with maximum spore production the final day (10); however, maximum spore production on lentils occurred on day 6 (2.28 × 10^6^ ± 2.78 × 10^5^ conidia mL^−1^) followed by a decrease in conidia, which accompanied a noticeable reduction in lentil size on day 8 and 10. Differences of pulse seeds compared to corn over day intervals for spore production (conidia mL^−1^) was significant for chickpeas on day 8 and 10 (*p* ≤ 0.01; Supplementary Table 1).

### 3.3 Aflatoxin content and GFP fluorescence

**Figure 5:**
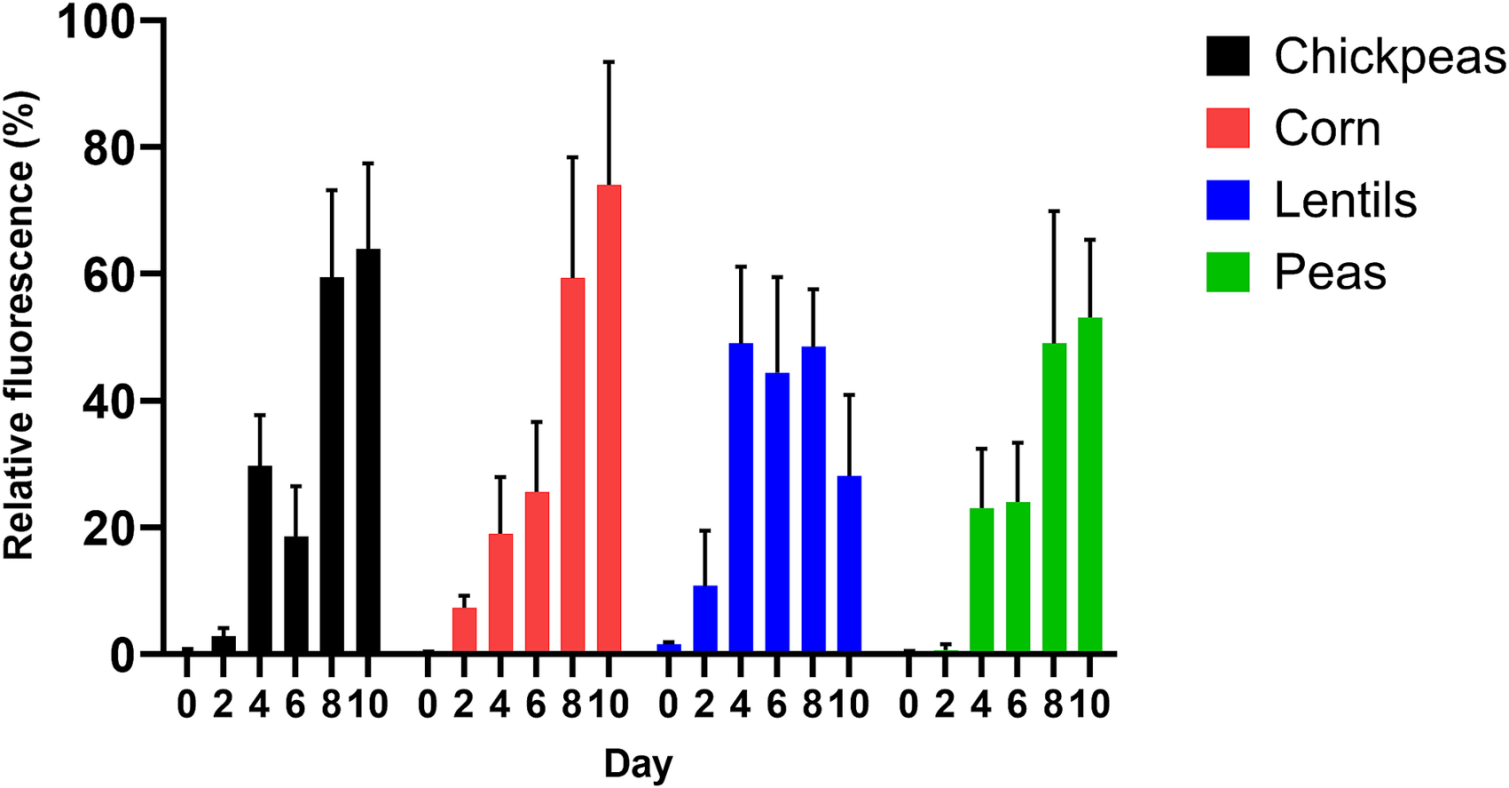
Production of AF 70-GFP on 2-day intervals after inoculation (as indicated by relative fluorescence %) on chickpeas, corn, lentils, and peas.

The amount of *A. flavus* fungal colonization by chickpeas, lentils, peas, and corn, as estimated by GFP fluorescence, was measured at the day intervals 0, 2, 4, 6, 8, and 10 after inoculation (Figure 5). Corn had the highest average GFP relative fluorescence and fungal colonization (30.93 ± 12.02%), with a maximum value of 74.04 ± 12.02% compared to the pulses (*p* = 0.63). Peas had the lowest average relative fluorescence (25.05 ± 9.26%) compared to corn (*p* = 0.35). Lentils had an average GFP relative fluorescence of 30.43 ± 8.36%, with a maximum value of 49.06 ± 8.36%; whereas chickpeas had an average GFP relative fluorescence of 29.18 ± 11.18%, with a maximum value of 63.92 ± 11.18%. Maximum GFP fluorescence was recorded for chickpeas, corn, and peas on day 10; however, lentils had the highest maximum value on day 8 (Figure 5; Supplementary Table 1).

**Figure 6:**
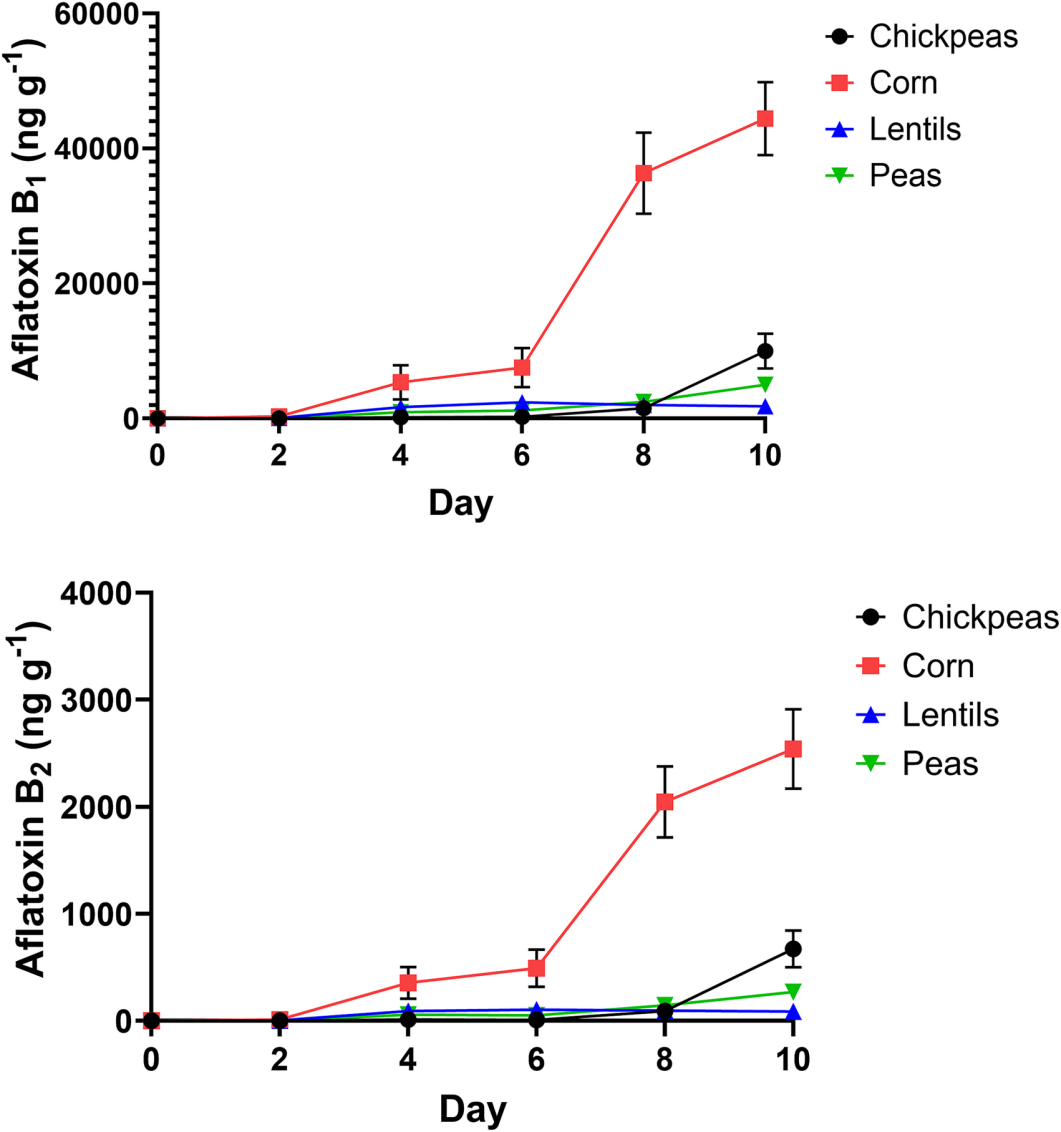
Growth of AFB_1_ and AFB_2_ (ng/g^−1^) on 2-day intervals after inoculation of chickpeas, corn, lentils, and peas.

AFB_1_ and AFB_2_ production in the four plant-rich protein sources was determined by UPLC (Figure 6). Corn was contaminated by the most AFB_1_ (15658.0 ± 7975.0 ng/g^−1^ or ppb), with a maximum value of 44428.0 ng/g^−1^ (Figure 6; Supplementary Table 1). Lentils were the least contaminated with average AFB_1_ (1325.0 ± 416.4 ng/g^−1^), with a maximum value of 2390.0 ng/g^−1^. On day 8 and 10, all pulses had statistically significant differences compared to corn for AFB_1_ and AFB_2_ (*p* < 0.001; Supplementary Table 1). Similarly, for AFB_2_, corn had the highest quantity (907.6 ± 449.6 ng/g^−1^), with a maximum value of 2540.0 ng/g^−1^, and lentils had the lowest average AFB_2_ (63.6 ± 19.6 ng/g^−1^)^,^ with a maximum value of 103.7 ng/g^−1^. Differences of pulses compared to corn over day intervals for AFB_1_ and AFB_2_ is in Supplementary Table 1.

**Figure 7:**
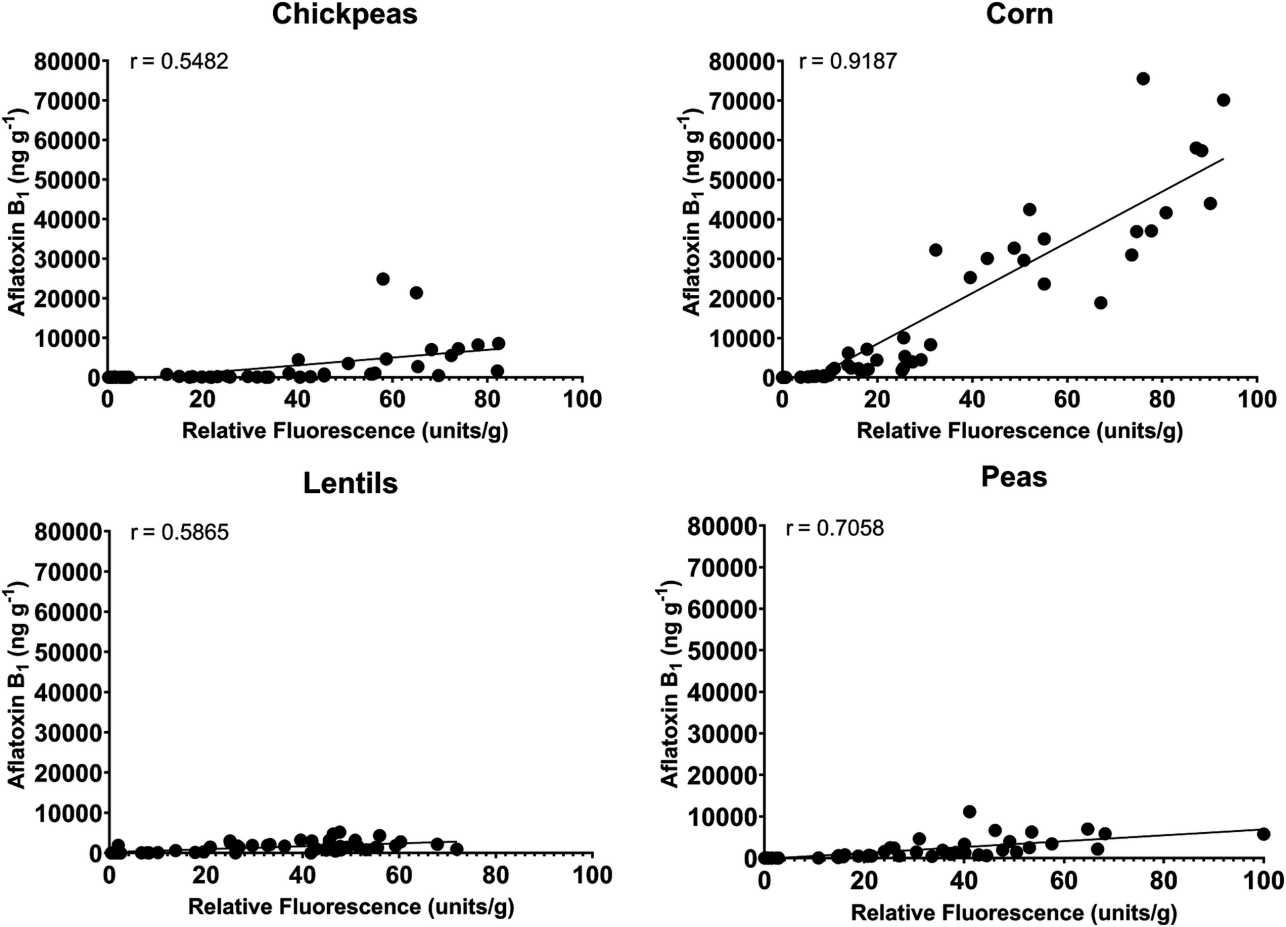
Correlation between relative fluorescence and AFB_1_ produced from AF 70-GFP for chickpeas, corn, lentils, and peas.

A correlation analysis between relative fluorescence and AFB_1_ production by AF-70 GFP was compared to determine the connection between fungal growth and aflatoxin production (Figure 7). There was a very strong positive correlation (*r* = 0.92) between relative fluorescence and AFB_1_ in corn. There was a strong positive correlation (*r* = 0.71) between relative fluorescence and AFB_1_ in peas. Additionally, there was a moderate positive correlation (*r* = 0.55 and *r* = 0.57) between relative fluorescence and AFB_1_ in chickpeas and lentils, respectively. *Aspergillus flavus* in corn (measured by AF-70 GFP) is a good indication of aflatoxin contamination, whereas infection of peas, and to a lesser extent chickpea and lentils infection, is not always a good indication of aflatoxin contamination.

**Figure 8:**
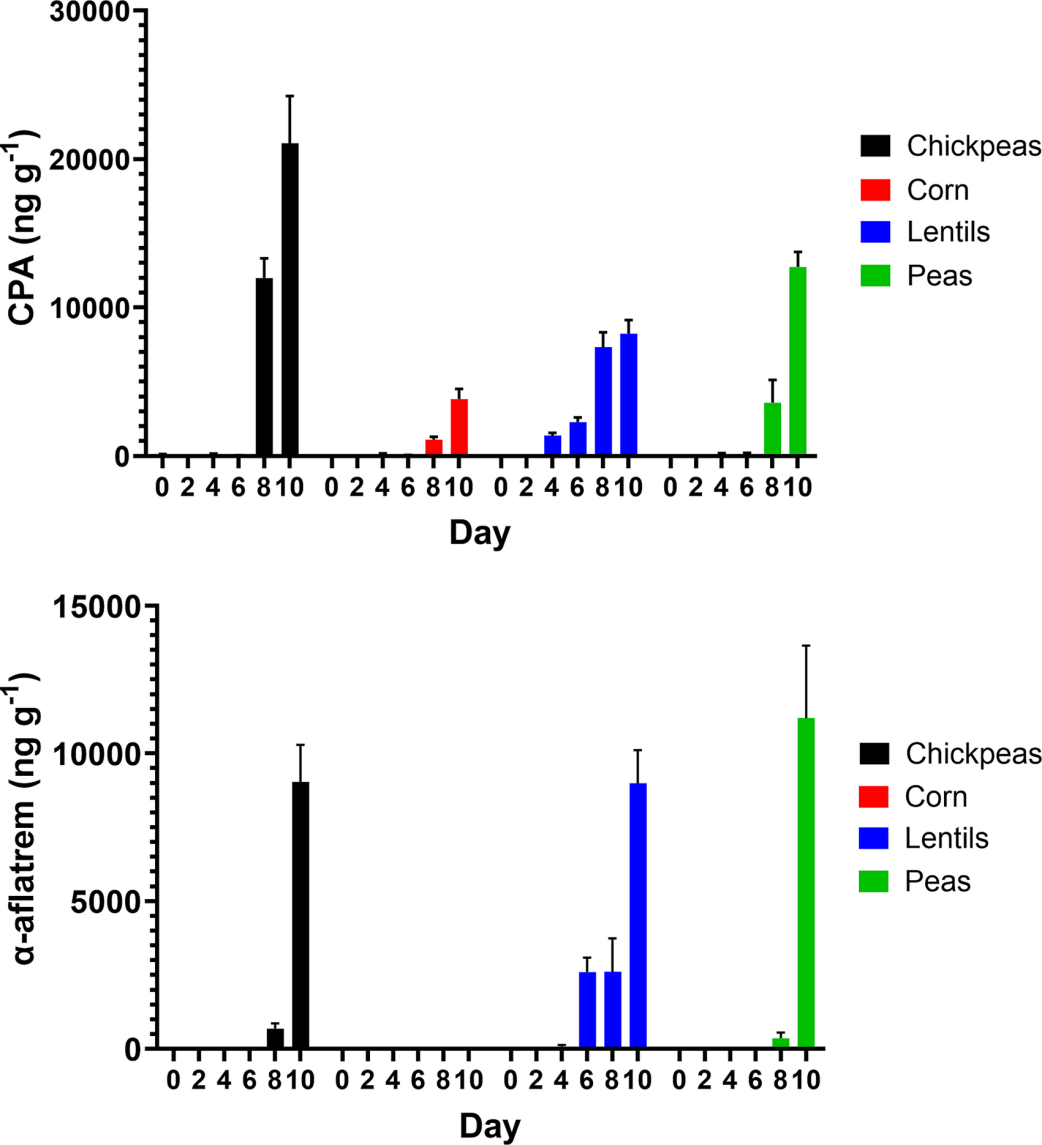
Production of CPA (ng/g^−1^) and α-aflatrem (ng/g^−1^) on 2-day intervals after inoculation on chickpeas, corn, lentils, and peas.

Cyclopiazonic acid (CPA), a phytotoxin with potential virulence factor, was measured across the four plant-rich protein sources determined by UPLC–MS. CPA production in corn had an average of 851.0 ± 621.30 ng/g^−1^. Chickpeas had the highest overall average at 5548.0 ± 3661.0 ng/g^−1^. Lentils averaged 3208.0 ± 1492.0 ng/g^−1^ for CPA, whereas, peas averaged 2768.0 ± 2074.0 ng/g^−1^, respectively. Additionally, α-aflatrem, a tremorgenic mycotoxin produced from *A. flavus*, was measured across the four plant-rich protein sources determined by UPLC–MS (Figure 8). Corn had the lowest average at 1.4 ± 0.9 ng/g^−1^ of α-aflatrem production. Lentils had the highest average with 2381.0 ± 1420.0 ng/g^−1^ of α-aflatrem production. Similarly, chickpeas averaged 1620.0 ± 1487.0 ng/g^−1^ whereas peas averaged 1926.0 ± 18550.0 ng/g^−1^ across all day intervals. Differences of pulses compared to corn over day intervals for α-aflatrem is in Supplementary Table 1.

## 4 Discussion

Pulses serve as a protein alternative or compliment to animal-based products (Henchion et al., 2017; Kunz et al., 2020; Quintieri et al., 2023). Increasingly, global citizens are incorporating pulses into their diet, driven by health interests and environmental sustainability awareness (Singh et al., 2024). The Food and Agriculture Organization of the United Nations (FAO) announced in 2016 it was the “Year of Pulses” to highlight their global importance, with consumption steadily increasing in recent decades, projecting to reach a $27 billion industry by 2030 (Singh et al., 2024). The benefits of using pulses in human diets are multifaceted, including improvement of cardiovascular health, enhanced metabolism, obesity prevention, and overall gut microbiome improvement due to their rich nutrient profile, including proteins, fiber, vitamin, and minerals (Curran, 2012; Mudryj et al., 2014; Patterson et al., 2009; Singh et al., 2024). Additionally, pulses are crucial for crop diversification, contributing to a resilient food chain. Ensuring the safety of pulses in food and feed is essential due to their global health relevance (Kunz et al., 2020). Therefore, in this study, growth of *A. flavus*, and production of aflatoxins, CPA, and α-aflatrem were examined using a KSA procedure over a 10-day timeframe to determine infection patterns, fungal growth, and colonization of undamaged pulse seeds (chickpeas, lentils, and peas) under a controlled environment, with cereal grain corn as a positive control.

Using an *A. flavus* strain expressing GFP genes to track aflatoxin contamination in pulses is a new method that has previously been applied to monitor fungal spread and aflatoxin levels in corn and cottonseed (Rajasekaran et al., 2008; Rajasekaran et al., 2013; Rajasekaran et al., 2017). This method is easy, sensitive, and rapid, allowing the evaluation of resistance or susceptibility of undamaged seeds based on GFP fluorescence. In corn, a strong correlation exists between GFP fluorescence and aflatoxin levels (Rajasekaran et al., 2013; Figure 6), with our study showing an exceptionally high correlation (*r* = 0.92). However, in pulses such as chickpeas, lentils, and peas, the direct relationship between *A. flavus* infection (Figure 7) and aflatoxin accumulation (Figure 6) was less robust, with correlations of *r* = 0.71 in peas, and lower correlations of *r* = 0.55 and 0.57 in chickpeas and lentils, respectively. This discrepancy is possibly due to the presence of antifungal factors in pulses, for example, interference from seed coat compounds like flavonoids, polyphenols, or differences in lipid, lipoxygenase, and oxylipin content (Doehlert et al., 1993; Burow et al., 1997; Xue et al., 2003). Minimal AFB_1_ and AFB_2_ production was observed in pulses compared to corn under identical conditions (Acuña-Gutiérrez et al., 2022; Davidson et al., 2012; Figures 4, 5). Moreover, in our study, the degradation of lentils, peas, and chickpeas during the cleaning process on days 8 and 10, likely due to complete internal tissue rot, may partly explain the reduced aflatoxin production despite high external spore counts and GFP fluorescence. Finally, it is important to note that GFP fluorescence primarily emanates from young *A. flavus* mycelia and spore structures, while older fungal structures such as sclerotia and spores exhibit diminished fluorescence. This characteristic, along with inherent differences in seed coat composition among pulses, contributes to the limitations of using GFP as a universal marker for fungal colonization in these diverse seed types.

Pulses have been shown to resist aflatoxin accumulation despite high fungal infection rates with *Aspergillus* spp. (Acuña-Gutiérrez et al., 2022; Figures 5–7). Specific characteristics of pulses, including thick and hard seed coats, antifungal proteins, high phenolic compounds, and oxylipins, contribute to their ability to regulate aflatoxin accumulation by protecting against phytopathogenic fungal attacks and reducing spore germination and/or mycelial growth (Acuña-Gutiérrez et al., 2022; Kralova et al., 2006; Makun et al., 2010; Martínez et al., 2017; Zabka and Pavela, 2013). The antifungal protein *pisumin*, identified in *Pisum sativum* varieties, has demonstrated efficacy against fungal pathogens such as *Alternaria*, potentially explaining the absence of its toxins in pea samples (Kralova et al., 2006). While direct research on *P. sativum* and *A. flavus* involving pisumin remains limited, other antifungal proteins, such as defensins, serve critical roles in plant immunity by disrupting fungal cell membranes and reducing infection severity. Seed coat structure further supports aflatoxin suppression, functioning as both a physical and biochemical barrier against fungal invasion. Thick seed coats, particularly in lentils, may contribute to reduced conidial production (1.60 × 10^6^ ± 3.04 × 10^5^ conidia mL^−1^), compared to chickpeas, which supported the highest levels of *A. flavus* conidial production (3.76 × 10^6^ ± 1.38 × 10^5^ conidia mL^−1^) (Supplementary Table 1). The delayed peak of conidia production in lentils (day 6) followed by a reduction on days 8 and 10 suggests a potential structural or biochemical factor influencing fungal development.

Phenolic compounds, secondary metabolites involved in plant defense, play a significant role in reducing aflatoxin accumulation. Flavonoids, tannins, and phenolic acids have been reported to exhibit antimycotoxigenic properties (Ahmed et al., 2022; Castano-Duque et al., 2022; Loi et al., 2020). While *in vitro* studies have demonstrated the effectiveness of phenolic extracts in reducing mycotoxin content across cereals, fruits, algae, and other plant products (Hua et al., 1999), research specifically on pulses remains limited. In this study, lentils, which exhibited lower *A. flavus* conidial production and fungal colonization (relative GFP fluorescence of 30.43 ± 8.36%), also had the lowest levels of aflatoxin contamination (1325.0 ± 416.4 ng/g^−1^ AFB_1_), highlighting the potential contribution of phenolic and structural defense mechanisms (Figure 6). Additionally, lipid-derived oxylipins, including oleic and linoleic acids, are key regulators of aflatoxin biosynthesis (Doehlert et al., 1993). The differential response of pulse crops to fungal colonization and aflatoxin accumulation suggests that variability in oxylipin content may be an underlying factor. For instance, lentils exhibited peak fungal colonization on day 8, followed by a reduction in conidia and seed shrinkage, which coincided with significantly lower aflatoxin levels compared to corn (*p* < 0.001; Supplementary Table 1). Further investigation into oxylipin profiles among protein-rich pulses is warranted to assess their role in aflatoxin suppression (Acuña-Gutiérrez et al., 2022; Cardador-Martinez et al., 2002; Singh, 2017).

AFB_1_ and AFB_2_ levels were inversely related to the production of CPA and α-aflatrem, other toxic *A. flavus* secondary metabolites, with higher levels observed in pulses compared to corn (Figure 8). CPA, produced by certain species of *Aspergillus* and *Penicillium* fungi, is found in various food sources including cereals, legumes, milk, meat, and cheese (Burdock and Flamm, 2000). The CPA levels measured in this study, particularly in chickpeas (5548.0 ± 3661.0 ng/g^−1^) and lentils (3208.0 ± 1492.0 ng/g^−1^), are significantly higher compared to corn (851.0 ± 621.30 ng/g^−1^) and align with previous studies reporting increased CPA production in legume-rich environments (Chang et al., 2009). CPA is a tremorgenic mycotoxin that causes symptoms such as weight loss, fever, diarrhea, dehydration, ataxia, immobility, and muscle spasms, primarily affecting the gastrointestinal tract, liver, spleen, and muscle tissues. Although CPA mycotoxicosis is considered less harmful compared to aflatoxin toxicity, its relatively high presence in pulses raises concerns regarding its potential health implications. Despite its toxicological significance, the FDA has not established regulatory guidelines for CPA, highlighting a gap in food safety assessments. However, the tolerable daily intake for CPA of 0.1 μg/kg body weight has been proposed by extrapolating toxicity data from test animals to humans (De Waal, 2002; Ostry et al., 2018). α-Aflatrem, another tremorgenic mycotoxin produced by *A. flavus* (Gallagher and Wilson, 1978), is a potent neurotoxin known to cause tremors and neurological disorders, such as mental confusion, seizures, and hyperexcitability, in rats and cattle (Valdes et al., 1985). Similarly, CPA in this study, pulses exhibited higher α-aflatrem concentrations compared to corn, with lentils having the highest levels (2381.0 ± 1420.0 ng/g^−1^) followed by peas (1926.0 ± 1855.0 ng/g^−1^) and chickpeas (1620.0 ± 1487.0 ng/g^−1^), while corn contained the lowest levels (1.4 ± 0.9 ng/g^−1^). α-Aflatrem is part of a diverse class of indole diterpene metabolites produced by *A. flavus* that concentrate in hardened fungal mycelia (i.e., sclerotia) (Gloer, 1995). These compounds function as antiinsectans, protecting sclerotia from predation. The production of α-aflatrem is regulated by the *veA* gene, which is also involved in the biosynthesis of aflatoxin and CPA (Duran et al., 2009). α-Aflatrem remains largely unregulated despite its neurotoxic effects. The observed inverse relationship between aflatoxin production and CPA/α-aflatrem levels suggests a potential shift in secondary metabolite biosynthesis regulation within *A. flavus*. This phenomenon may be linked to metabolic competition for biosynthetic precursors and regulatory pathways influenced by global transcription factors such as veA and laeA (Calvo et al., 2004; Cary et al., 2015). These genes are known to coordinate secondary metabolite production and may play a role in prioritizing the synthesis of CPA and α-aflatrem in certain environmental conditions. Understanding this regulatory interplay could provide insight into fungal secondary metabolism and help evaluate whether pulses, despite their lower aflatoxin contamination, may still pose significant food safety risks due to elevated CPA and α-aflatrem levels.

Addressing the issue of aflatoxin in pulses requires a thorough understanding of their growth conditions, storage stability, and post-harvest handling techniques to implement preventative measures globally. Evaluating aflatoxin infection in chickpeas, lentils, peas, and potentially other protein-rich pulses like beans is essential for making informed decisions regarding health and safety risks (Singh et al., 2024). Understanding the growth of aflatoxins in pulses is crucial for developing effective risk analysis techniques that can be translated into political decisions and regulatory compliance worldwide. Employing good agricultural practices through a holistic approach with regular monitoring can significantly reduce the risk of aflatoxin in pulses, through improved analysis and measurement (Nyangi and Runyogote, 2024). Awareness of these factors across the entire supply chain is vital for maintaining food and feed safety in the coming years, given the projected population increase and the need for sustainable food sources to reduce the carbon footprint (Gräfenhahn and Beyrer, 2024). Future research will focus on evaluating the impact of *A. flavus* growth in both fresh and powdered plant products, as well as assessing the stability of proteins during growth and storage. Moreover, a standardized methodology for *A. flavus* growth in pulses will be developed to enhance consistency in research and safety protocols. By adopting a comprehensive strategy that includes good agricultural practices, continuous monitoring, and effective risk management, the risk of aflatoxin contamination in pulses can be greatly reduced, ensuring food safety and security on a global scale.

## 5 Conclusion

Pulses, including chickpeas [*Cicer arietinum* L.], lentils [*Lens culinaris* Medik], and peas [*Pisum sativum* L.], are highly nutritious and protein-dense food products that are anticipated to gain popularity in the coming decades due to global demand. Despite increased usage in human diets, limited data has been published regarding *A. flavus* and aflatoxin infection in these pulses from a food and feed safety standpoint. This study presents a novel methodology to determine aflatoxin accumulation using a GFP-expressing *A. flavus* (AF-70 GFP) strain to assess fungal growth, and consequently aflatoxin infection rates, CPA, and α-aflatrem in chickpeas, lentils, and peas. Pulses did not exhibit higher amounts of aflatoxins compared to corn and other grains; however, CPA and α-aflatrem production was higher in pulses. Considering the health relevance of pulses for human and livestock diets in present day and in the future for sustainable aspects, more attention should be drawn to understanding growth conditions of aflatoxin in pulses in field, including growing conditions, to storage. Similarly, the mechanism of aflatoxin accumulation and resistance to fungal attacks, needs to be further explored. Aflatoxin contamination of pulses *in planta* and in stored, powdered commercial products will be examined in a subsequent study.

## Data Availability

The raw data supporting the conclusions of this article will be made available by the authors, without undue reservation.
