# Supplementary material for: Evaluation of aflatoxin contamination in protein-rich pulses using a GFP-expressing Aspergillus flavus strain
Source: Front Microbiol. 2025 May 29;16:1587035. doi: 10.3389/fmicb.2025.1587035 (PMC12159027; doi:10.3389/fmicb.2025.1587035)
Supplement: Supplementary file 1 [file Table_1.docx]

**Supplementary Table 1.** Summary statistics (mean + SEM) for chickpeas, lentils, peas, and corn by day.

| **Day** | **Seed Type** | **Aflatoxin B_1_ (ng/g^-1^)** | **Aflatoxin B_2_ (ng/g^-1^)** | **Spores (conidia/ml^-1^)** | **GFP^1^ Relative Fluorescence (%)** |
| --- | --- | --- | --- | --- | --- |
| 0 | Chickpea | 5.13 ± 4.52^3ns^ | 0.89 ± 0.79^ns^ | **^.^** | 0.57 ± 0.08^*^ |
| 0 | Lentil | 3.58 ± 0.86^ns^ | 0.26 ± 0.06^**^ | **^.^** | 1.55 ± 0.11^***^ |
| 0 | Pea | 3.33 ± 3.33^ns^ | 1.09 ± 1.09^ns^ | **^.^** | 0.36 ± 0.04^ns^ |
| 0 | Corn | 2.60 ± 0.43 | ND^4^ | **.** | 0.24 ± 0.07 |
| 2 | Chickpea | 0.72 ± 0.67^**^ | 0.09 ± 0.06^**^ | 4.85×10^5^ ± 1.11×10^5ns^ | 2.82 ± 0.43^***^ |
| 2 | Lentil | 89.66 ± 30.61^*^ | 3.71 ± 1.19^*^ | 5.24×10^5^ ± 5.84×10^4ns^ | 10.81 ± 2.89^ns^ |
| 2 | Pea | 1.04 ± 1.033^**^ | 0.62 ± 0.62^**^ | 2.86×10^5^ ± 4.96×10^4ns^ | 0.66 ± 0.30^***^ |
| 2 | Corn | 301.53 ± 52.51 | 12.83 ± 2.35 | 5.92×10^5^ ± 1.01×10^5^ | 7.34 ± 0.64 |
| 4 | Chickpea | 180.82 ± 105.32^ns^ | 12.01 ± 7.77^ns^ | 2.56×10^6^ ± 3.73×10^5*^ | 29.77 ± 2.65^*^ |
| 4 | Lentil | 1,675.61 ± 416.97^ns^ | 91.84 ± 25.39^ns^ | 1.39×10^6^ ± 2.66×10^5ns^ | 49.06 ± 4.03^***^ |
| 4 | Pea | 937.91 ± 309.57^ns^ | 57.22 ± 21.52^ns^ | 7.00×10^6^ ± 1.56×10^5ns^ | 23.08 ± 3.12^ns^ |
| 4 | Corn | 5,351.56 ± 2,537.94 | 355.09 ± 147.37 | 1.20×10^7^ ± 2.70×10^5^ | 19.00 ± 3.00 |
| 6 | Chickpea | 245.30 ± 68.15^ns^ | 8.48 ± 2.92^ns^ | 1.87×10^7^ ± 3.58×10^5ns^ | 18.53 ± 2.66^ns^ |
| 6 | Lentil | 2,389.90 ± 455.03^ns^ | 103.66 ± 21.66^ns^ | 2.28×10^7^ ± 2.78×10^5ns^ | 44.45 ± 5.01^*^ |
| 6 | Pea | 1,198.98 ± 332.14^ns^ | 41.11 ± 18.99^ns^ | 1.93×10^7^ ± 4.67×10^5ns^ | 24.02 ± 3.12^ns^ |
| 6 | Corn | 7,539.78 ± 2,887.84 | 491.91 ± 173.95 | 1.76×10^7^ ± 1.61×10^5^ | 25.58 ± 3.69 |
| 8 | Chickpea | 1,509.52 ± 563.46^**^ | 91.68 ± 46.94^***^ | 6.26×10^7^ ± 5.96×10^5***^ | 59.49 ± 4.58^ns^ |
| 8 | Lentil | 1,996.91 ± 451.51^**^ | 94.53 ± 21.12^***^ | 1.95×10^7^ ± 3.11×10^5ns^ | 48.53 ± 3.01^ns^ |
| 8 | Pea | 2,444.99 ± 777.19^**^ | 144.82 ± 58.64^**^ | 2.86×10^7^ ± 4.74×10^5ns^ | 49.05 ± 6.95^ns^ |
| 8 | Corn | 36,325.52 ± 6,009.65 | 2,045.76 ± 333.78 | 3.18×10^7^ ± 4.20×10^5^ | 59.35 ± 6.34 |
| 10 | Chickpea | 9,980.09 ± 2,566.83^**^ | 671.07 ± 172.19^**^ | 7.73×10^7^ ± 9.62×10^5***^ | 63.92 ± 4.50^ns^ |
| 10 | Lentil | 1,795.26 ± 237.69^**^ | 87.46 ± 15.37^***^ | 1.86×10^7^ ± 2.40×10^5**^ | 28.15 ± 4.27^***^ |
| 10 | Pea | 5,010.94 ± 1,001.82^**^ | 269.88 ± 61.31^***^ | 4.04×10^7^ ± 6.25×10^5ns^ | 53.12 ± 4.09^ns^ |
| 10 | Corn | 44,428.30 ± 5,427.44 | 2,540.28 ± 370.73 | 3.78×10^7^ ± 2.79×10^5^ | 74.04 ± 6.85 |

^1^Green fluorescent protein

^2^Cyclopiazonic acid

^3^Standard error of the mean (SEM)

^4^Not-detected

^*^Indicates level of Significance based on APA (* *p*<0.1), ** (*p*<0.05), *** (*p*<0.001), or NS for non-significant compared to corn as a control)

**Supplementary Table 1 Continued.** Summary statistics (mean + SEM) for chickpeas, lentils, peas, and corn by day.

| **Day** | **Seed Type** | **CPA^2^ (ng/g^-1^)** | **α-aflatrem (ng/g^-1^)** |
| --- | --- | --- | --- |
| 0 | Chickpea | 78.89 ± 55.08^ns^ | ND |
| 0 | Lentil | 4.78 ± 3.98^ns^ | ND |
| 0 | Pea | 8.67 ± 8.67^ns^ | ND |
| 0 | Corn | 14.33 ± 9.81 | ND |
| 2 | Chickpea | 5.44 ± 3.64^ns^ | ND |
| 2 | Lentil | 20.67 ± 14.11^ns^ | ND |
| 2 | Pea | ND^4ns^ | ND |
| 2 | Corn | 27.89 ± 12.00 | ND |
| 4 | Chickpea | 125.33 ± 32.56^ns^ | ND |
| 4 | Lentil | 1,378.33 ± 186.61^***^ | 82.89 ± 43.36 |
| 4 | Pea | 142.67 ± 57.17^ns^ | ND |
| 4 | Corn | 85.78 ± 85.78 | ND |
| 6 | Chickpea | 36.11 ± 22.94^ns^ | ND |
| 6 | Lentil | 2,286.56 ± 314.85^***^ | 2,603.89 ± 487.65 |
| 6 | Pea | 133.89 ± 74.46^ns^ | ND |
| 6 | Corn | 35.89 ± 34.41 | ND |
| 8 | Chickpea | 11,992.44 ± 1,305.03^***^ | 687.56 ± 178.49^*^ |
| 8 | Lentil | 7,325.78 ± 1,009.63^***^ | 2,605.22 ± 1,140.20^ns^ |
| 8 | Pea | 3,592.78 ± 1,532.31^ns^ | 361.11 ± 197.54^ns^ |
| 8 | Corn | 1,110.33 ± 178.93 | 5.11 ± 5.11 |
| 10 | Chickpea | 21,047.67 ± 3,196.33^**^ | 9,035.11 ± 1,252.77^***^ |
| 10 | Lentil | 8,232.78 ± 922.97^**^ | 8,996.89 ± 1,113.66^***^ |
| 10 | Pea | 12,730.11 ± 1,003.91^***^ | 11,193.67 ± 2,455.44^**^ |
| 10 | Corn | 3,831.78 ± 679.08 | 3.00 ± 2.44 |

^1^Green fluorescent protein

^2^Cyclopiazonic acid

^3^Standard error of the mean (SEM)

^4^Not-detected

^*^Indicates level of Significance based on APA (* *p*<0.1), ** (*p*<0.05), *** (*p*<0.001), or NS for non-significant compared to corn as a control)
